# Eight-Year Retrospective Analysis of Mortality in Patients with Moderate to Severe Hyponatremia: A Comprehensive Study

**DOI:** 10.3390/jcm14217834

**Published:** 2025-11-04

**Authors:** Yasemin Coşkun Yavuz, Zeynep Biyik, Muslu Kazım Korez, Mustafa Zahid Kaya, Lutfullah Altintepe

**Affiliations:** 1 Nephrology Department, Faculty of Medicine, Selçuk University, 42250 Konya, Türkiye; drzeynepbiyik@gmail.com (Z.B.); laltintepe@gmail.com (L.A.); 2 Biostatistics Department, Faculty of Medicine, Selçuk University, 42250 Konya, Türkiye; mkkorez@gmail.com; 3 Internal Medicine Department, Faculty of Medicine, Selçuk University, 42250 Konya, Türkiye; d.zahidkaya@gmail.com

**Keywords:** diuretics, echocardiography, heart failure, hyponatremia, mortality, nephrology

## Abstract

**Background/Objectives:** Hyponatremia is defined as a serum sodium concentration below 135 mEq/L. It is associated with increased morbidity and mortality. This study aimed to determine the factors associated with mortality in patients hospitalized with moderate to severe hyponatremia in the nephrology clinic and nephrology intensive care unit during an eight-year follow-up period. **Methods**: This retrospective study included patients admitted between January 2018 and October 2025 who were hospitalized due to moderate or severe hyponatremia. **Results**: Of 4270 patients, 337 (7.8%) were hospitalized with moderate to severe hyponatremia. The majority of patients were female (60.2%; n = 203). 242 patients (71.8%) had severe hyponatremia. The most common presenting complaint was nausea and vomiting, the most common month and season of presentation was July-Summer, and the most common cause of hyponatremia was drug-induced hyponatremia. The mortality rate was 40.7% (n = 137). The most common cause of death was decompensated heart failure. Factors independently affecting mortality; age (HR = 1.018, 95% CI 1.001–1.037, *p* = 0.047), malignancy (HR = 2.397, 95% CI 1.459–3.939, *p* < 0.001), number of hospitalizations (HR = 0.377, 95% CI 0.228–0.623, *p* < 0.001), EF (HR = 0.972, 95% CI 0.956–0.988, *p* < 0.001), high phosphorus (HR = 2.397, 95% CI 1.527–3.764, *p* < 0.001), furosemide use (HR = 1.638, 95% CI 1.018–2.636, *p* = 0.042) and fluid restriction. **Conclusions**: Advanced age, malignancy, high phosphorus levels, furosemide use, and fluid restriction were associated with increased mortality, whereas higher ejection fraction and greater number of hospitalizations were protective. These findings emphasize the importance of individualized management strategies and close follow-up in patients with moderate to severe hyponatremia.

## 1. Introduction

Hyponatremia is defined as a serum sodium concentration below 135 mEq/L [1]. Its prevalence in hospitalized patients ranges from 15 to 30% [2], and is even higher in the elderly population. Hyponatremia is associated with increased morbidity and mortality. Its etiology is broad. Syndrome of inappropriate antidiuretic hormone secretion (SIADH), cirrhosis, renal failure, heart failure, medications, and many other causes can lead to hyponatremia. Diagnosis is crucial because treatment is based on the underlying cause [3]. Acute severe hyponatremia can lead to seizures, disorientation, psychosis, coma, and even death, whereas chronic and mild hyponatremia presents with symptoms such as nausea, vomiting, headache, and fatigue [4].

The primary parameters used to determine the etiology of hyponatremia are volume status, serum and urine osmolality, and urine sodium levels. Additional tests may be necessary for differential diagnosis after these initial assessments [5].

Hyponatremia is classified based on serum sodium levels as mild (130–134 mEq/L), moderate (125–130 mEq/L), and severe (<125 mEq/L) [6]. In a retrospective study by Hao et al. on a general patient population, mortality was found to be higher in patients with hyponatremia compared to those without it. Furthermore, as the severity of hyponatremia increased, so did mortality [7]. Similarly, in a large cohort from Denmark, hospitalized patients with hyponatremia had significantly higher mortality compared to those with normal sodium levels [8]. In the said study involving over 50,000 patients, every 1 mmol/L decrease in serum sodium was associated with a 2.3% increase in mortality [9]. Besides mortality, even a slight reduction in plasma sodium was associated with morbidities such as frailty, attention deficits, sarcopenia, osteoporosis, and cardiac fibrosis [5].

In the etiology of hyponatremia, SIADH, heart failure, and medications are among the top causes [10]. The most common cause is typically the SIADH [11].

In this study, at eight years, the etiologies, comorbidities, laboratory findings, season and month of admission, and treatments of patients hospitalized in our clinic due to hyponatremia were compared with their mortality outcomes.

## 2. Methods

**Figure 1:**
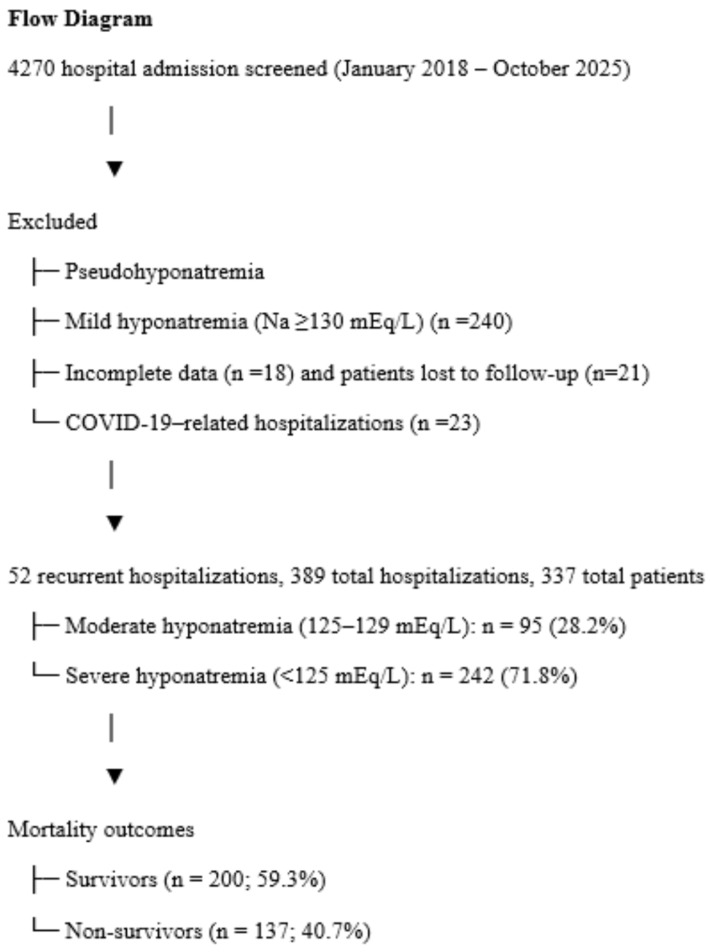
Flow diagram of study.

This retrospective cohort study was conducted at the Department of Nephrology, Selçuk University Faculty of Medicine, Konya, Türkiye, between January 2018 and October 2025. The hospital is a tertiary referral center for nephrology cases. Approved by Selçuk University Ethics Committee (No: 2024/18). Informed consent waived due to retrospective design. All procedures followed the Declaration of Helsinki). Data from patient records, including demographic information, comorbidities, medications, treatments received during hospitalization, potential causes of hyponatremia, and laboratory findings, were collected. Mortality data from this eight-year period were retrieved from the national health data registry system (e-Nabiz) of the Ministry of Health of the Republic of Türkiye. Additionally, sodium levels at admission and discharge, season and month of admission, the number of hospitalizations due to hyponatremia, and the duration of hospital stays were recorded. We included in our study retrospectively those who were hospitalized in our clinic for 8 years. Since patients with pseudohyponatremia were already excluded before hospitalization, none of the patients we included in our study were pseudohyponatremic. Exclusion criteria included pseudohyponatremia, mild hyponatremia (Na ≥ 130 mEq/L), missing data, patients lost to follow-up and COVID-19-related hospitalizations (Figure 1).

Sodium values corrected based on the patients’ blood glucose measurements were calculated. For every 100 mg/dL increase in serum glucose, 2.4 mEq/L was added to the serum sodium [12]. Patients with corrected sodium values within the normal range according to hyperglycemia were excluded from the study.

Patients’ comorbidities including diabetes mellitus (DM), hypertension, heart failure, coronary arter disease (CAD), chronic kidney disease (CKD), chronic obstructive pulmonary disease (COPD), malignancy, presence of pneumonia during hospitalization were questioned and recorded.

Medication use was categorized into diuretics (thiazide and thiazide-like, potassium-sparing, and loop diuretics), antipsychotics, antidepressants, anticonvulsants, angiotensin converting enzyme inhibitor, angiotensin receptor blocker (ACE inhibitors/ARBs), proton pump inhibitor (PPI), chemotherapeutic agents, and other medications.

In clinically euvolemic patients with a serum osmolality below 275 mosm/kg H_2_O, urine osmolality above 100 mosm/kg, urine sodium of 30 mEq/L, and normal thyroid function tests and cortisol levels, the etiology of hyponatremia was considered to be SIADH. In addition, urinary sodium above 30 mEq/L was considered positive for renal sodium losses [13,14].

Complete blood count analyses were performed using a Sysmex XN-300 hematology analyzer(Sysmex, Hamburg, Germany). Biochemical parameters, thyroid stimuling hormon (TSH), morning cortisol level (08:00 h), adrenocorticotrophic hormon (ACTH), serum and urine osmolality, and urine sodium were determined with Roche cobas e801 chemistry, freeze-point osmometer and Rocheimmunoassay systems via electrochemiluminesansce method (Roche Diagnostics, Mannheim, Germany). Estimated glomerular filtration rate (eGFR) was calculated and given by the laboratory according to the CKD-EPI formula based on serum creatinine.

Additionally, ejection fraction (EF) and pulmonary artery pressure (PAP) values were recorded for patients who had echocardiography within the last 1 year.

Data from living and deceased patients over the eight-year period were compared to identify factors affecting mortality.

Patients due to COVID-19 were excluded from the study.

### 2.1. Power Analysis

The sample size was calculated to detect a medium effect (Cohen’s d = 0.5) with a two-sided α = 0.05 and 99% power, requiring at least 148 participants per group.

### 2.2. Statistical Analysis

All statistical analyses were conducted using the statistical software language *R* version 4.1.2 (The *R* Foundation for Statistical Computing, Vienna, Austria; https://www.r-project.org). Before the analyses, the normality of the data was assessed using the Shapiro-Wilk test and Q-Q plots, while the homogeneity of group variances was assessed using the Levene’s test. The findings for numerical variables included in the study were presented as mean ± standard deviation or median with quartiles [1st quartile–3rd quartile], and categorical variables were presented as frequency (*n*) and percentage. The presence of statistically significant differences in demographic and clinical characteristics, as well as laboratory findings between moderate and severe hyponatremia patients, was evaluated using independent sample *t*-test, Welch’s I-test, or Mann–Whitney *U* test. Age, comorbidities, antihypertensive medication use, and the use of hypertonic sodium, fluid restriction, and tolvaptan in treatment were compared among patient groups using Fisher’s exact test and Yates’s correction for continuity (Yates’s chi-squared test). These tests were also used to compare the characteristics of deceased and discharged patients. We used Cox proportional hazard regression model to determine prognostic factors for mortality. Hazard ratios (HR) were presented with 95% confidence intervals. The significance level was taken as 5%.

## 3. Results

The files of 4270 patients hospitalized in the nephrology clinic over approximately eight years were reviewed. There were 52 recurrent hospitalizations, and the total number of hospitalizations was 389. There were 337 patients hospitalized for moderate to severe hyponatremia (7.8%). Hyponatremia was severe in 242 patients (71.8%). 60.2% (n = 203) of the patients were female. The mean age was 68.1 ± 15.2 years.

The month with the highest number of hospitalizations was July, and the season with the highest number of hospitalizations was summer (*p* value, respectively *p* < 0.001 and *p* = 0.0002).

The mean follow-up period was 590.8 ± 597.7 days (min 1, max 3305 days).

240 patients were excluded from the study due to mild hyponatremia, 18 patients due to incomplete data, 21 patients due to loss of follow-up, and 23 patients with hyponatremia due to COVID-19 positivity.

The most common presenting complaints were nausea and vomiting in 84 patients (25%), weakness in 53 patients (15.8%), dyspnea in 50 patients (14.9%), and confusion in 34 patients (10.1%). The number of patients without any complaints was 20 (6%).

Among comorbidities, hypertension was the most common and was present in 247 patients (73.3%). DM was present in 138 patients (40.8%), CKD in 80 patients (23.7%), CAD in 145 patients (43%), malignancy in 66 patients (19.6%), COPD in 82 patients (24.3%), and pneumonia in 26 patients (7.7%).

The most common causes of hyponatremia in patients were drug-related hyponatremia in 122 patients (36.2%), hypervolemic hyponatremia in 89 patients (27.2%), and SIADH in 74 patients (22.6%).

A total of 337 patients hospitalized with moderate to severe hyponatremia were included in the study, of whom 137 (40.7%) died. The most common causes of death were decompensated heart failure in 52 patients (37.9%), cancer in 33 patients (24%), and sepsis in 27 patients (19.7%).

### 3.1. Mortality Comparison (Table 1)

#### Demographic and Clinical Characteristics

The mean age of non-survivors was significantly higher than that of survivors (71.7 ± 14.1 vs. 65.8 ± 15.6 years, *p* < 0.001). The distribution of sex did not differ significantly between the groups (*p* = 0.139).

**Table 1:** Comparison of Demographic, Clinical, Laboratory, and Treatment Characteristics Between Survivors and Non-Survivors.

	Mortality		
	Survivors (*n* = 200)	Non-Survivors (*n* = 137)	*p*-Value
Demographical			
Age (years)	65.78 ± 15.64	71.66 ± 14.09	<**0.001** ^1^
Sex (F/M)	127/73	76/61	0.139 ^2^
Admission season			0.999 ^2^
Autumn	47 (23.5)	33 824.1)	
Spring	40 (20)	27 (19.7)	
Summer	70 (35)	48 (35)	
Winter	43 (21.5)	29 (21.2)	
Comorbidities			
Diabetes mellitus	80 (40)	58 (42.3)	0.668 ^2^
Hypertension	150 (75)	97 (70.8)	0.392 ^2^
CKD	44 (22)	36 (26.3)	0.365 ^2^
CAD	77 (38.5)	68 (49.6)	**0.043 ** ^2^
Malignity	22 (11)	44 (32.4)	<**0.001** ^2^
COPD	49 (24.5)	33 (24.1)	0.931 ^2^
Pneumonia	11 (5.5)	15 (10.9)	0.102 ^3^
No. of hospitalization	1.96 (0 to 18)	1.18 (1 to 3)	**0.005 ** ^4^
Length of stay (days)	6.67 (1 to 25)	9.73 (1 to 43)	<**0.001** ^4^
EF	58 (18 to 68)	55 (10 to 65)	<**0.001** ^4^
PAP	32.5 (18 to 72)	40 (20 to 73)	0.086 ^4^
Drugs			
Thiazide diuretic use	82 (41)	46 (33.6)	0.168 ^2^
Antidepressive-antiepileptic	59 (29.5)	36 (26.3)	0.518 ^2^
ACEi/ARB use	97 (48.5)	62 (45.3)	0.558 ^2^
Spiranolactone	27 (13.5)	41 (29.9)	<**0.001** ^2^
Furosemide	27 (13.6)	47 (34.3)	<**0.001** ^2^
Chemotherapy	16 (8)	30 (21.9)	<0.001 ^2^
Other drugs	171 (85.5)	118 (86.1)	0.871 ^2^
Glucose (72–106 mg/dL)	118 [97–141.25]	121 [100–158]	0.282 ^4^
High glucose	143 (71.5)	102 (74.5)	0.550 ^2^
Urea nitrogen (16.6–48.5 mg/dL)	42 [24.5–84]	60 [37–130]	<**0.001** ^4^
High urea	86 (43)	81 (59.1)	**0.004 ** ^2^
Creatinine (0.5–0.9 mg/dL)	1.02 [0.70–1.87]	1.25 [0.84–2.32]	**0.015 ** ^4^
High creatinine	98 (49)	85 (62)	**0.018 ** ^2^
eGFR (ml/min/1.73 m^2^)	72.55 [32.35–97.40]	55 [28–87.5]	**0.011 ** ^4^
Admission Sodium (135–145 mEq/L)	122 [117.75–125]	122 [118–126]	0.603 ^4^
Discharge Sodium (135–145 mEq/L)	134 [132–136]	134 [132–137]	0.775 ^4^
Low discharge sodium	115 (57.5)	74 (54)	0.527 ^2^
Potassium (3.5–5.1 mmol/L)	4.27 [3.79–4.88]	4.49 [3.94–5.30]	**0.007 ** ^4^
High potassium	54 (27)	57 (41.6)	**0.005 ** ^2^
Calcium (8.6–10.5 mg/dL)	8.80 [8.38–9.30]	8.80 [8.20–9.20]	0.310 ^5^
Low calcium	67 (33.5)	59 (43.1)	0.075 ^2^
Magnesium (1.62.6 mg/dL)	1.74 [1.57–1.95]	1.84 [1.61–2.11]	**0.027 ** ^4^
Low magnesium	83 (41.5)	57 (41.6)	0.985 ^2^
Phosphorus (2.5–4.5 mg/dL)	3.30 [2.90–4.30]	3.50 [2.90–3.80]	0.090 ^4^
High phosphorus	42 (21)	43 (31.4)	**0.031 ** ^2^
Albumin (3.5–5.2 gr/dL)	3.76 ± 0.55	3.40 ± 0.63	<**0.001** ^1^
Low albumin	81 (40.5)	75 (54.7)	**0.010 ** ^2^
Uric acid (2.4–5.7 mg/dL)	5.25 [3.38–7.23]	6.30 [4.10–8.20]	**0.005 ** ^4^
High uric acid	90 (45)	72 (52.6)	0.173 ^2^
TSH (0.27–4.2 µIU/mL)	1.36 [0.81–2.28]	1.48 [0.80–2.33]	0.870 ^4^
Cortisol (6.2–19.4 µg(dL)	15.10 [11.40–19.70]	15.45 [12–20.08]	0.471 ^4^
ACTH (7.2–63.3 pg/mL)	25.50 [14.07–45.88]	24.80 [15–43.60]	0.968 ^4^
Urine osmolality (50–1200 mosm/kgH_2_O)	382.75 ± 150.82	394.20 ± 164.96	0.721 ^1^
Urine sodium (mEq/L)	49.50 [30–70.25]	49 [29–88]	0.443 ^4^
High urine sodium (>20 mEq/L)	43 (26.2)	25 (26.9)	0.908 ^2^
Serum osmolality (275–285 mosm/kgH_2_O)	270 [265–278]	273 [259–290]	0.362 ^4^
Treatment			
High hypertonic sodium	161 (80.5)	100 (73)	0.105 ^2^
Fluid restriction	84 (42)	84 (61.3)	<**0.001** ^2^
Tolvaptan	14 (7)	9 (6.6)	>0.999 ^3^

^1^ student’s *t*-test; ^2^ Pearson chi-square test; ^3^ Chi-square with Yates continuity correction; ^4^ Mann-Whitney *U* test; ^5^ student’s *t*-test. Abbreviations: CKD, chronic kidney disease; CAD, coronary artery disease; COPD, chronic obstructive pulmonary disease; PAP, pulmonary artery pressure; EF, ejection fraction. *p* < 0.05 are written in bold.

### 3.2. Comorbidities

Among comorbid conditions, the prevalence of coronary artery disease (CAD) (*p* = 0.043) and malignancy (*p* < 0.001) was significantly higher in non-survivors. Other chronic comorbidities such as diabetes mellitus (DM), hypertension, chronic kidney disease (CKD), chronic obstructive pulmonary disease (COPD), and pneumonia did not differ significantly between groups (*p* > 0.05).

### 3.3. Hospitalization Parameters

Non-survivors had a longer length of hospital stay (median = 9.7 days, range 1–43) compared to survivors (median = 6.7 days, range 1–25) (*p* < 0.001). The number of previous hospitalizations was also lower among non-survivors (*p* = 0.005).

### 3.4. Medication Use

The use of aldosterone antagonists (spironolactone) (*p* < 0.001), furosemide (*p* < 0.001), and chemotherapy (*p* < 0.001) was significantly more frequent in non-survivors. No significant differences were observed regarding thiazide diuretics, anticonvulsants, ACE inhibitors/ARBs, or other medications (*p* > 0.05).

### 3.5. Laboratory Findings

Non-survivors had significantly higher serum urea (*p* < 0.001), creatinine (*p* = 0.015), potassium (*p* = 0.007), magnesium (*p* = 0.027), uric acid (*p* = 0.005*), and phosphorus (*p* = 0.031) levels, and lower estimated glomerular filtration rate (eGFR) (*p* = 0.011) and serum albumin concentrations (*p* < 0.001).

Categorical analysis showed that the proportion of patients with elevated urea (59.1% vs. 43.0%; *p* = 0.004), elevated creatinine (62.0% vs. 49.0%; *p* = 0.018), elevated potassium (41.6% vs. 27.0%; *p* = 0.005), elevated phosphorus (31.4% vs. 21.0%; *p* = 0.031), and low albumin (54.7% vs. 40.5%; *p* = 0.010) was significantly greater among non-survivors.

Admission and discharge sodium levels did not differ significantly between survivors and non-survivors (*p* > 0.05). Similarly, no significant group differences were observed for serum calcium, TSH, cortisol, ACTH, urine or serum osmolality (*p* > 0.05).

### 3.6. Treatment Characteristics

Fluid restriction was applied significantly more often in non-survivors than in survivors (61.3% vs. 42.0%; *p* < 0.001). The frequency of hypertonic saline use and tolvaptan treatment did not differ significantly between groups (*p* > 0.05).

In the univariate Cox regression analysis, increased age increased the risk of death (HR = 1.021, 95% CI 1.008–1.034, *p* < 0.001). Among comorbidities, the presence of coronary artery disease (CAD) (HR = 1.461, 95% CI 1.045–2.044, *p* = 0.026) and malignancy (HR = 2.685, 95% CI 1.865–3.864, *p* < 0.001) were significantly related to higher mortality. A lower number of hospitalizations (HR = 0.642, 95% CI 0.472–0.874, *p* = 0.004) and reduced ejection fraction (EF) (HR = 0.99, 95% CI 0.955–0.983, *p* < 0.001) were also associated with increased mortality risk (Table 2).

Regarding laboratory parameters, elevated urea (HR = 1.005, *p* < 0.001), creatinine (HR = 1.089, *p* = 0.007), phosphorus (HR = 1.675, *p* = 0.005), potassium (HR = 1.267, *p* < 0.001), and uric acid (HR = 1.073, *p* = 0.008) levels were significantly associated with higher mortality, whereas lower albumin levels also increased the risk (HR = 1.532, *p* = 0.013). In addition, use of furosemide (HR = 2.228, *p* < 0.001) and fluid restriction (HR = 1.960, *p* < 0.001) were predictors of poor prognosis (Table 2).

**Figure 2:**
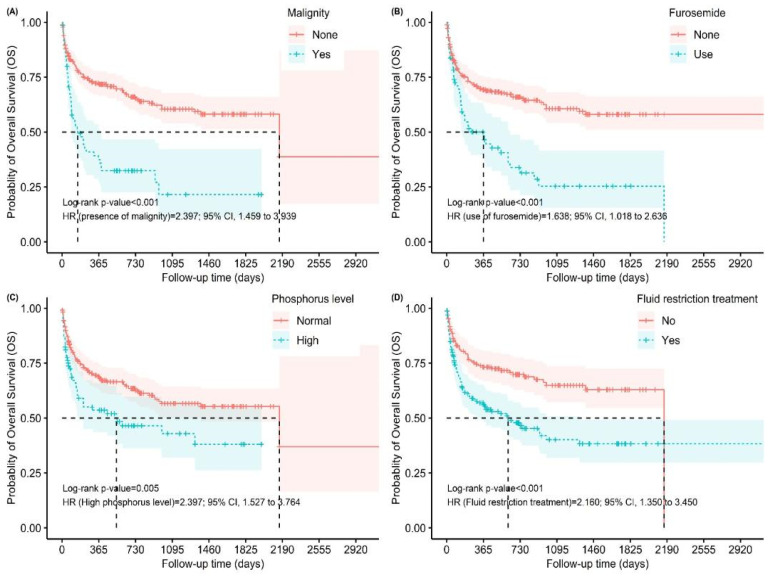
Kaplan–Meier Curves for Overall Survival According to Clinical and Biochemical Factors. Panels show overall survival stratified by (**A**) presence of malignancy, (**B**) use of furosemide, (**C**) serum phosphorus level, and (**D**) fluid restriction therapy. Shaded areas indicate 95% confidence intervals. The number of patients at risk is shown below each curve.

In the multivariate model, after adjustment for confounding factors, age (HR = 1.018, 95% CI 1.001–1.037, *p* = 0.047), malignancy (HR = 2.397, 95% CI 1.459–3.939, *p* < 0.001), number of hospitalizations (HR = 0.377, 95% CI 0.228–0.623, *p* < 0.001), EF (HR = 0.972, 95% CI 0.956–0.988, *p* < 0.001), high phosphorus (HR = 2.397, 95% CI 1.527–3.764, *p* < 0.001), furosemide use (HR = 1.638, 95% CI 1.018–2.636, *p* = 0.042), and fluid restriction (HR = 2.160, 95% CI 1.350–3.450, *p* = 0.001) remained independent predictors of mortality (Table 2, Figure 2).

## 4. Discussion

There are many causes of hyponatremia, with the most common being known as SIADH [15,16]. In this study, the three most common causes identified drug-related hyponatremia, hypervolemic hyponatremia and SIADH. In patients with hyponatremia, the etiology does not always involve a single cause. These patients usually have multiple comorbidities and use several medications. Therefore, it is not easy to determine the etiology precisely. A study involving patients with severe hyponatremia found that 44% of patients had multifactorial etiology [17]. We evaluated the patient’s complaint, physical examination findings and laboratory findings together and evaluated the ‘most probable’ cause as the cause of hyponatremia. However, even in medication-induced cases, other accompanying factors (such as drugs causing SIADH) might also have contributed to hyponatremia. A 10-year pharmacovigilance study by Ramirez et al. also showed that drugs are the most common cause of severe hyponatremia [18]. The majority of our patient group had severe hyponatremia. In a 5-year retrospective study published by Becerre Anez and colleagues in 2025, thiazide-associated hyponatremia was the most common hyponatremia [19]. In this respect, our study parallels these studies. The reason why drug-related hyponatremia was the most common cause in these two recent studies and in our study may be that the development of patient data and record systems in recent years, the medications used by patients can be easily accessed, and the tendency to use thiazide as part of antihypertensive treatment has increased.

Additionally, hypervolemic hyponatremia was the second most common cause in our study. This may be due to the habit in our center of referring patients with right or left heart failure to Nephrology when there is concomitant renal function loss or electrolyte disturbance. This approach is not limited to our center [20]. In the study conducted by Ternero-Vega and colleagues in Spain, where they evaluated hyponatremic patients according to their volume status, hypervolemic hyponatremia was seen most frequently, although the rates were close to each other [21].

Although hyponatremia is known to increase mortality, the number of studies focusing solely on patients with hyponatremia is quite limited. In a study by Mustajoki et al., which included patients with severe hyponatremia (serum Na < 116 mEq/L) presenting to the emergency department, the 1-year mortality was found to be 18% [17]. Another retrospective study reported a 1-year mortality of 22% in severe hyponatremia [22]. In our study, we found the mortality rate to be 40.7%. The longer follow-up period (8 years) in our study might be a possible reason for the higher mortality rate. Furthermore, it is unlikely that moderate-severe hyponatremia will occur alone in a patient without comorbidities or multiple medication use. The high prevalence of comorbidities affecting many vital organs, such as hypertension, DM, CAD, CKD, COPD, and malignancy in our patient group, may be a contributing factor to the high mortality rate.

In this study, the causes of death were decompensated heart failure in 52 patients (37.9%), cancer in 33 patients (24%), and sepsis in 27 patients (19.7%). Hyponatremia is known to be an important mortality indicator in decompensated heart failure. In the meta-analysis by Zhao et al., it was shown that 1-year mortality was higher in patients with heart failure [23]. We could not find a study showing the causes of long-term mortality in patients hospitalized primarily with hyponatremia. However, we can assume that the relationship between heart failure mortality and hyponatremia is two-way.

There are studies suggesting that mortality increases as the severity of hyponatremia increases [24,25]. However, the effect of lower sodium levels and more severe hyponatremia on mortality is not always linear or independent. For example, in a large Danish cohort, no greater mortality was found in the sodium <120 mmol/L group than expected [8]. In our study, we did not find a relationship between the severity of hyponatremia and mortality. Al Yaqoubi et al., like us, did not find a relationship between 1-year mortality and severity of hyponatremia [26]. They considered the reason as single-center. It is probably not sodium level alone but the underlying disease severity and comorbidity burden that stand out as the main factors driving mortality.

In this study, the month with the highest number of hospitalizations was July, and the season with the highest number of hospitalizations was summer. This is also consistent with studies in the literature [27,28].

The clinical symptoms of hyponatremia typically result from brain edema. Early symptoms include nausea and vomiting, while late symptoms include lethargy, seizures, stupor, and coma [29]. In our study, the most common presenting complaints of patients were nausea and vomiting.

In this study, we found that mortality was significantly higher in patients with CAD, one of the comorbidities. Additionally, deceased patients had lower ejection fraction. In the meta-analysis of the Meta-Analysis Global Group in Chronic heart failure (MAGGIC) involving patients with preserved EF and reduced EF, both preserved and reduced EF patients with hyponatremia had higher in-hospital mortality [30]. Similarly, another study in France found that low sodium levels in heart failure patients were associated with increased mortality [31]. In a study evaluating in-hospital mortality in hyponatremic patients, mortality was found to be higher in patients with hypervolemic hyponatremia and heart failure [32]. In our study, mortality was also higher in this patient group after 8 years of follow-up. The rate of furosemide use was higher in our patients who died during follow-up. The fact that the profile of deceased patients included those with heart failure, and the higher use of furosemid in these patients is considered a possible reason for this outcome. In this study, when multiple Cox regression analysis was performed, we found that furosemide use was still associated with mortality. In our study, mortality was more common in patients who underwent fluid restriction. The patients who underwent fluid restriction were hypervolemic and had malignant SIADH. Therefore, the relationship between mortality and the patients’ hypervolemia and malignancy, rather than the fluid restriction itself, is more likely to be related to mortality.

We found that malignancy was also a mortality-related factor in patients hospitalized with hyponatremia. This is an expected finding and has been supported by studies [22,33].

In this study, we found hyperphosphatemia to be an independent predictor of increased mortality in the moderate and severe hyponatremia population. Phosphorus intake can be affected by many factors, including loss of renal function, nutrition, malignancy, and medications. The patients in our study had a high number of comorbidities and, therefore, were taking multiple medications. Shuto et al. demonstrated in their study that elevated phosphorus levels cause endothelial damage [34]. In a study conducted in patients with subarachnoid hemorrhage, hyperphosphatemia was also found to be associated with increased mortality [35]. In a recent study of a large cohort of patients undergoing cardiac surgery, hyperphosphatemia was significantly associated with prolonged intubation, prolonged hospital stay, and increased hospital mortality [36].

The limitations of our study are its single-center design and retrospective nature. We are of the opinion that our study is significant as it focuses solely on hyponatremic patients and evaluates clinical and laboratory parameters effective on mortality of hyponatremic patients over a long follow-up period. The majority of the current studies compare hyponatremic patients with non-hyponatremic patients from general patient population. We only included patients with moderate to severe hyponatremia who were hospitalized and examined and treated. This study examines the long-term follow-up of hyponatremic patients from the perspective and approach of a nephrologist. Furthermore, its comprehensive analysis of the patients’ medications, including the season and month of admission, is a key feature of this study. Also, our follow up period is long. We believe that these are an advantage for our study.

## 5. Conclusions

In conclusion, we have confirmed that hyponatremia is more common in older age and female gender. We can say that medications have come to the forefront in this study, along with recent studies on the etiology of hyponatremia. Furthermore, we can say that advanced age, heart failure, and malignancy are the leading causes of mortality in these patients. We should not ignore the contribution of high phosphorus levels to mortality. These theories should be supported by more and more comprehensive studies on hyponatremia, the most common electrolyte disorder.

## Figures and Tables

**Table 2:** Univariate and Multiple Cox Proportional Hazards Models Identifying Independent Predictors of In-Hospital Mortality.

	Univariate Model		Multiple Model	
	HR [95% CI]	*p*-Value	HR [95% CI]	*p*-Value
Demographical				
Age (years)	1.021 [1.008 to 1.034]	<0.001	1.027 [1.006 to 1.049]	0.009
Comorbidities				
CAD	1.461 [1.045 to 2.044]	0.026	1.171 [0.644 to 2.129]	0.603
Malignity	2.685 [1.865 to 3.864]	<0.001	2.183 [1.238 to 3.848]	0.007
Volume status (ref:euovolemic)				
Hypervolemic	2.121 [1.454 to 3.092]	<0.001	1.027 [0.559 to 1.887]	0.930
Hypovolemic	1.154 [0.728 to 1.829]	0.542	1.315 [0.695 to 2.488]	0.398
No. of hospitalization	0.642 [0.472 to 0.8739	0.004	0.334 [0.191 to 0.582]	<0.001
Length of stay (days)	1.070 [1.044 to 1.096]	<0.001	1.021 [0.989 to 1.054]	0.189
EF	0.99 [0.955 to 0.983]	<0.001	0.978 [0.959 to 0.997]	0.024
Drugs				
Aldactone	1.937 [1.342 to 2.794]	<0.001	1.216 [0.694 to 2.129]	0.493
Furosemide	2.228 [1.562 to 3.179]	<0.001	1.789 [1.039 to 3.082]	0.035
Laboratory				
Urea	1.005 [1.002 to 1.007]	<0.001		
High urea	1.732 [1.230 to 2.437]	0.001	0.508 [0.274 to 0.942]	0.031
Creatinine	1.089 [1.023 to 1.159]	0.007		
High creatinine	0.647 [0.458 to 0.915]	0.013	0.886 [0.359 to 2.186]	0.793
eGFR	0.993 [0.989 to 0.998]	0.008	2.506 [1.118 to 5.613]	0.025
Potassium	1.267 [1.101 to 1.458]	<0.001		
High potassium	1.583 [1.126 to 2.224]	0.008	1.241 [0.773 to 1.992]	0.371
Magnesium	1.129 [0.899 to 1.417]	0.296		
High phosphorus	1.675 [1.166 to 2.408]	0.005	2.242 [1.317 to 3.815]	0.003
Albumin	0.462 [0.652 to 0.607]	<0.001		
Low albumin	1.532 [1.094 to 2.145]	0.013	1.381 [0.857 to 2.226]	0.185
Uric acid	1.073 [1.018 to 1.130]	0.008	0.704 [0.430 to 1.153]	0.163
Treatment				
Fluid restriction	1.960 [1.387 to 2.768]	<0.001	0.404 [0.209 to 0.777]	0.007

## Data Availability

Dataset splitting upon request from authors.
